# The Business Man in Dentistry

**Published:** 1904-06

**Authors:** 


### THE BUSINESS MAN IN DENTISTRY.

It has been frequently said that professional men are poor business men. It may with equal truth be declared that a business man rarely makes a good professional man.

The metropolis is ever the Mecca towards which avaricious eyes are turned. Fortunes exist and have been made in New York, and the great majority of the discontented throughout the country are eager for an opportunity to walk Broadway or Wall street in search of the golden nuggets with which imagination strews those thoroughfares. What wonder that the provincial dentist, earning a paltry five thousand a year, should desire to establish himself in New York and earn some of those fabulous fees that may so easily be wrung from the millionaires in exchange for amalgam fillings in their molars? None! The only wonder would be that any could succeed in such a venture. The effort has been made by several during the past decade, and it has been of interest to note the methods and progress of at least one or two. It seemed remarkable that any dentist should abandon a practice already established and begin over, merely because rumor states that a limited few—a very limited few—earn a considerable number of thousands annually in this city. It seemed questionable whether, along ethical lines, a practice which would be at all lucrative could be obtained within a period of five years by one who was an utter stranger. This supposition, however, overlooked the instincts of the business man. The story of one such man, the details of whose metropolitan career have come under observation, may be instructive.

It is just about five years since he first became one of the several thousand dentists of this section. It is doubtful whether he has achieved that fortune for which he sought, yet he has received at least a few of the larger fees of which he had dreamed. At first he opened a modest office in a good neighborhood and then went to live at a boarding house. This brought a few acquaintances, and by adroitly leading conversations in the dining room into dental channels, with an occasional recitation of the newer dental achievements being accomplished, the deduction being, of course, that the relator was proficient in these more advanced dental operations, attention was directed toward himself. Well managed, such hints attracted patients, and so soon as the mine was thoroughly worked it was always possible to move to a new boarding house, there to find renewed possibilities. Then there were the haunts of men, the cafes attached to the larger hotels, and the chance to become acquainted with the clerks therein. Dental services rendered for such as these, at reduced rates, rendered obligations, payable by the recommending of guests in the hotels, some of whom, unlike the habitues of the boarding houses, had money.

Now it happened that this particular business man was not lacking in skill. He could fill teeth well, regulate teeth well, do crown and bridgework above the average, and in all ways was sufficiently well equipped to have successfully managed a permanent practice. What he lacked was the real professional instinct. He was a dentist, true, but he was preeminently a business man. His chief aim was the acquirement of money. Strangely enough, New Yorkers, especially those that pay large fees, are far from stupid. They quickly discover whether a dentist is working for them or for himself. So it came to pass that when this business dentist for large fees would fill teeth with porcelain, when gold would have served better for less money, the bill was paid, but the patient sought another dentist when next in need. Then there were those who submitted to extensive bridgework affixed to teeth that were so loose they should have been extracted. The fees were large, but when the abscesses arrived and the bridges, with the roots, were removed, there were other dentists who did the extracting and restoration of the denture. And so with several other unusual or novel operations which patients were persuaded to accept once. They accepted them but once, then looked elsewhere for a dentist. So it has come to pass that it is no uncommon occurrence to have a new patient say: "I am ashamed to acknowledge that I have been in the hands of that 'fakir,' Dr. ———. He fooled me once, but I have had my lesson."

Thus we find that it is possible to be too much of a business man when essaying to practice a profession; and the moral of the tale is, that perhaps it might pay better in the end for a professional man to be a professional man.—*Editorial Items of Interest.*

